# Role of the Amygdala in Antidepressant Effects on Hippocampal Cell Proliferation and Survival and on Depression-like Behavior in the Rat

**DOI:** 10.1371/journal.pone.0008618

**Published:** 2010-01-08

**Authors:** Jorge E. Castro, Emilio Varea, Cristina Márquez, Maria Isabel Cordero, Guillaume Poirier, Carmen Sandi

**Affiliations:** Laboratory of Behavioral Genetics, Brain Mind Institute, École Polytechnique Fédérale de Lausanne (EPFL), Lausanne, Switzerland; Centre de Recherches su la Cognition Animale - Centre National de la Recherche Scientifique and Université Paul Sabatier, France

## Abstract

The stimulation of adult hippocampal neurogenesis by antidepressants has been associated with multiple molecular pathways, but the potential influence exerted by other brain areas has received much less attention. The basolateral complex of the amygdala (BLA), a region involved in anxiety and a site of action of antidepressants, has been implicated in both basal and stress-induced changes in neural plasticity in the dentate gyrus. We investigated here whether the BLA modulates the effects of the SSRI antidepressant fluoxetine on hippocampal cell proliferation and survival in relation to a behavioral index of depression-like behavior (forced swim test). We used a lesion approach targeting the BLA along with a chronic treatment with fluoxetine, and monitored basal anxiety levels given the important role of this behavioral trait in the progress of depression. Chronic fluoxetine treatment had a positive effect on hippocampal cell survival only when the BLA was lesioned. Anxiety was related to hippocampal cell survival in opposite ways in sham- and BLA-lesioned animals (i.e., negatively in sham- and positively in BLA-lesioned animals). Both BLA lesions and low anxiety were critical factors to enable a negative relationship between cell proliferation and depression-like behavior. Therefore, our study highlights a role for the amygdala on fluoxetine-stimulated cell survival and on the establishment of a link between cell proliferation and depression-like behavior. It also reveals an important modulatory role for anxiety on cell proliferation involving both BLA-dependent and –independent mechanisms. Our findings underscore the amygdala as a potential target to modulate antidepressants' action in hippocampal neurogenesis and in their link to depression-like behaviors.

## Introduction

The enhancement of adult hippocampal neurogenesis induced by a wide variety of antidepressant treatments has attracted a great deal of attention [1], [2], [3]. In rodents, chronic antidepressant administration has been shown to increase both the proliferation of neural progenitors in the subgranular zone of the dentate gyrus (DG) [4] and the survival of these newborn neurons [5]. Intensive research is devoted to unravel the neurobiological mechanisms involved in the effects of antidepressants [6], [7], and to disentangle how antidepressant effects on depression-like behavior may be mediated by hippocampal neurogenesis [8], [9]. The search for mechanisms of action has mostly focused on the involvement of molecular pathways, including the activation of specific serotonin receptors [9], [10], [11], the cAMP-CREB signaling pathway [12], and neurotrophins, particularly brain-derived neurotrophic factor (BDNF), fibroblast growth factor (FGF-2) and vascular endothelial growth factor (VEGF) [13]. Although less explored, network activity also seems to be critical for the neurogenic effects of antidepressants [3]. Local hippocampal activity has been shown to affect adult hippocampal neurogenesis at different phases, from cell proliferation to cell maturation and integration [14], [15], [16]. However, the possibility that antidepressant effects on adult hippocampal neurogenesis are influenced by the concerted action of other brain regions has not been, to our knowledge, as yet explored.

The amygdala appears to be an excellent candidate to modulate antidepressant-related hippocampal neurogenesis. Substantial evidence indicates that the amygdala is a site of action of antidepressants [17], [18], [19], [20], [21], [22]. Selective serotonin reuptake inhibitors (SSRI) antidepressant treatment was shown to modulate amygdala responses directly in humans without requiring a clinical change in mood or initial amygdala pathology, while diminishing the perception of fear [23], [24]. In depressed subjects, decreased amygdala volume [25] and increased amygdala response to masked emotional faces [26], [27] were normalized after chronic antidepressant (in particular, SSRI) treatment. In rodents, SSRI treatment resulted in reduced levels of fear conditioning, which depends critically on amygdala function [28]. In addition to its well known role in the mediation of emotions [29], [30], the amygdala [particularly its basolateral division (BLA)] has been critically implicated in the emotional potentiation of memory by facilitating information processing and storage in other structures, notably the hippocampus [31], [32], [33]. Moreover, activation of the BLA was shown to regulate neural plasticity in the DG [34], [35], the former being also associated with stress-induced deficits in hippocampal long-term potentiation (LTP) and spatial memory in rats [36], [37]. Furthermore, animal studies have also shown that acute electrical stimulation of the amygdala (i.e., kindling) increases hippocampal neurogenesis [38], [39], [40], [41].

Here, we investigated whether the amygdala and anxiety contribute to the effects of antidepressants on hippocampal cell proliferation and survival, and on a behavioral index of depression-like behavior (the forced swim test). We used a lesion approach targeting the basolateral amygdala together with a chronic treatment with the SSRI antidepressant fluoxetine. Anxiety (as indexed by animal's anxiety levels before the onset of antidepressant treatment) was included in the analyses given its important modulatory role in both vulnerability and recovery from depression [42]. Moreover, given the interest for understanding how indices of both cell proliferation and survival, as well as of depression-like behavior relate to each other [3], [8], [9], [43], the relationship between these factors was also explored. We applied parametric statistics followed by structural equation modeling (SEM) to test for unique and shared variance of the factors on depression-like behavior, as well as to assess how cell proliferation and survival are engaged by the interacting effects of fluoxetine, BLA lesions, and anxiety, in relation to the selected index of depressive-like behavior.

## Materials and Methods

### Subjects

Adult male Sprague-Dawley rats (Charles River Laboratories, France) were used (250–275 g of body weight at the beginning of the experiment, approximately three months old). Rats were housed two per cage and maintained with *ad libitum* access to food under light (12 hours light/dark cycle; lights on at 7:00 AM) and temperature (22±2°C) controlled conditions. Experimental procedures were approved by the Cantonal Veterinary Authorities (Vaud, Switzerland).

### General Experimental Procedures

**Figure 1:**
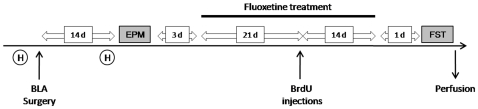
Schematic plan of experimental procedures and timetable. After handling (H), rats were submitted to stereotaxic surgery for BLA or sham lesions. After recovery, animals were handled again and levels of anxiety were characterized using the Elevated Plus Maze (EPM). Next, chronic fluoxetine treatment was initiated and BrdU was injected after twenty one days of drug administration. After interruption of fluoxetine treatment, depression-like behavior was evaluated using the Forced Swim Test (FST) and then sacrificed.

Rats were submitted to stereotaxic surgery for Sham (n = 38) or BLA lesions (n = 43). After recovery from surgery (11 days), rats were handled again for 3 days and tested in the elevated plus-maze (EPM) to determine individual anxiety levels. Three days after testing in the EPM, Sham and BLA lesion groups were each divided into two subgroups that were matched according to anxiety levels and body weight, and assigned to the drug treatment ‘vehicle’ or ‘fluoxetine’. Therefore, the study comprised four groups: Sham lesion/Vehicle (Sh/Vh), Sham lesion/Fluoxetine (Sh/Flx), BLA lesion/Vehicle (BLA/Vh), and BLA lesion/Fluoxetine (BLA/Flx). Fluoxetine treatment was given daily during 5 weeks. Since the number of animals in each group was corrected after histological verification of the extent of the lesions, the sample size for each group differed from the initial group size and is reported in the corresponding Results section. Bromodeoxyuridine (BrdU) injections to evaluate survival of proliferating cells were given on days 21 and 22 of fluoxetine treatment. One day after the end of fluoxetine treatment, rats were tested in the forced-swim test and sacrificed 15 min after the test (see Figure 1).

### Elevated Plus Maze

Anxiety levels were evaluated using the EPM test [44]. Briefly, the test consists of two opposing open arms (45×10 cm) perpendicular to two enclosed arms (45×10×50 cm) that extend from a central platform (10×10 cm), elevated 65 cm above the floor. The rats were placed individually on the central platform and allowed to explore the maze for five minutes. Their behavior was monitored using a video camera and analyzed with a computerized tracking system (Ethovision 3.1.16, Noldus IT, The Netherlands). The time spent in the open and closed arms, distance moved and transitions between the different arms were recorded.

### Surgery

Rats were anesthetized using an intraperitoneal injection of a combination of 75 mg/kg of ketamine chlorhydrate (2-(2-chlorophényl)-2-méthylamino-cyclohexan-1-one; Graeub, Bern, Switzerland) and 10 mg/kg of xylazine chlorhydrate (N-(2,6-dimethylphenyl)-5,6-dihydro-4H-1,3-thiazin-2-amine; Graeub, Bern, Switzerland). Rats were then placed into a stereotaxic apparatus (Stoelting, USA), the scalp was incised and retracted, the head position was adjusted to place Bregma and Lambda in the same horizontal plane and small burr holes were drilled in the skull above the basolateral complex of the amygdala to accommodate a 28 gauge infusion cannula (2.5 mm posterior to bregma, +/− 4.5 mm lateral to bregma, 8.2 mm ventral to the skull surface). A 10 µl Hamilton syringe mounted on an infusion pump (Harvard Apparatus, South Natick, MA) and connected to the cannula with polyethylene tubing was used to deliver infusions. BLA lesions were made by injecting bilaterally 0.4 µl of NMDA (N-methyl-D-aspartic acid) (20 µg/µl; Sigma, St. Louis, MO) in 100 mM PBS, pH 7.4, at a rate of 0.2 µl/min. Sham lesion rats underwent an identical procedure, except that the cannula was filled with vehicle, lowered and no infusions were made [45]. After surgery, animals were returned to their home cages and allowed 11 days of recovery followed by three days of handling before behavioral testing.

### Chronic fluoxetine treatment

Fluoxetine hydrochloride ((7)-N-methyl-g-(4-[trifluoromethyl]-phenoxy)-benzenepropanamine) (Mepha, Aesch, Switzerland) was administered daily during 35 days in the rats' tap water (10 mg/kg) in their drinking bottles [21]. Body weight and fluid intake were recorded regularly. The drug concentration was adjusted according to changes in water intake and body weight in order to reach the target dose of 10 mg/kg across the experiment. Vehicle groups received tap water during the same period. In order to control for water intake and thus fluoxetine dosage, a Plexiglas separator allowing visual and olfactory contact divided each housing cage into two parts.

### BrdU injections

Rats were injected with the thymidine analog bromodeoxyuridine (BrdU; 50 mg/kg, i.p.) (Sigma-Aldrich, St Louis, MO, USA) dissolved in a phosphate buffer (0.1 M, pH 8.4). Four injections were made, one every 12 hours over a period of two days, starting on day 21 of fluoxetine treatment [46].

### Forced-swim Test

One day after the last fluoxetine treatment, rats were submitted to a forced-swim test to evaluate depression-like behaviors [47], [48]. Briefly, they were individually placed in a plastic beaker (25 cm diameter, 46 cm deep) containing 30 cm of water (25±1°C) for 5 minutes. Even though this test is conventionally performed in two sessions, 24 hours apart, during which time spent in immobility is scored [49], we only performed one testing session. The reasons for applying a single forced swim session are the following: (i) The standard forced swimming test was developed for its sensitivity to evince behavioral effects of wide range of clinically active antidepressant drugs that, when injected on day 1 result in reduced immobility on the test performed on day 2 [49]. However, when the test has been used to assess whether chronic stress can affect behavioral “despair” and, hence, depression-like behavior, a single session has proven sensitive to evidence increased behavioral immobility in depressed animals [50], [51]. (ii) The second important reason not to extend the protocol to a second forced swim test taking place on a subsequent day was related to the main goal of the study, aiming to compare the impact of different factors (notably, amygdala lesions and fluoxetine treatment) on hippocampal cell proliferation. Since hippocampal cell proliferation and/or survival have been shown to be affected by prior stress [52], [53], we reasoned that sacrificing animals immediately after a single stress session (forced swim) would not allow sufficient time for the stress-induced changes in cell proliferation to evolve.

Behavior was recorded with a video camera and the time spent immobile (making only those movements necessary to keep the snout above the water), swimming, climbing or diving was quantified manually using a computer program (The Observer 5.0.25, Noldus IT, 2003).

### Histological procedures

Fifteen minutes after the end of the forced-swim test, animals were anesthetized with isofluorane and transcardially perfused using 0.9% saline solution followed by a fixative solution of paraformaldehyde 4% in phosphate buffered saline (PBS, pH = 7.5). After perfusion-fixation, brains were removed and post-fixed in the same solution for four hours. Next, 50 µm coronal sections were cut on a vibratome (VT 1000S; Leica, Glattbrugg, Switzerland) and stored in 4°C PBS. Cell proliferation in the dentate gyrus was measured by assaying the expression of the endogenous marker, Ki67. Survival of proliferating cells was assayed by staining for BrdU.

Alternate series of 1 in 10 sections were processed “free-floating” for immunohistochemical visualization of newborn neurons (Ki67) and mature neurons (NeuN), the latter helping determine the extent of the lesions. Briefly, sections were incubated with 3% H_2_O_2_ in PBS for 10 minutes to block endogenous peroxidase activity. After washing in PBS (3×10 minutes), the sections were treated for 1 hour with 5% normal donkey serum (NDS, Jackson ImmunoResearch Laboratories, West Grove, PA) in PBS with 0.2% Triton-X100 (Sigma-Aldrich, St. Louis, MO). After washing in PBS, they were incubated overnight at room temperature with monoclonal mouse anti-Ki67 antibody (1∶200; Novocastra) or monoclonal mouse anti-NeuN biotinylated antibody (1∶200, Chemicon). PBS containing 0.2% Triton-X100 and 3% NDS (PBST) was used for primary and secondary antibodies dilution. After washing in PBS, sections for Ki67 were incubated for 120 minutes with donkey anti-mouse IgG, (1∶250; Jackson ImmunoResearch Laboratories). Sections for NeuN did not require secondary antibody because the antibody used was biotinylated. After washing in PBS, sections were incubated during one hour with avidin-biotin-peroxidase complex (ABC; A 1∶200, B 1∶200; Vector Laboratories, Peterborough, UK), prepared 30 minutes before in PBS. And after washing in PBS, color development was achieved by incubating with 3,3′ diaminobenzidine tetrahydrochloride (DAB; 0.5 mg/ml Sigma-Aldrich) for 15 minutes.

For BrdU immunohistochemistry, a previous step of denaturation was performed: after blockade of the endogenous peroxidase, sections were incubated for 1 hour at 60°C, treated with 2 N HCl (30 min at 37°C) and then rinsed in borate buffer during 5 min (0.1 M; pH 8.4). After washing in PBS (3×10 minutes), the sections were treated for 1 hour with 5% normal donkey serum (NDS, Jackson ImmunoResearch Laboratories, West Grove, PA) in PBS with 0.2% Triton-X100 (Sigma-Aldrich, St. Louis, MO). After washing in PBS, they were incubated overnight at room temperature with a mouse monoclonal anti-BrdU antibody (1∶500, Oxford). Following washes in PBS, sections were incubated for 120 minutes with donkey anti-rat IgG biotinilated antibody. Sections were processed in parallel, and immunoreactivities were visualized by the biotin–streptavidin technique (ABC kit; Vector Laboratories, Peterborough, UK) using 3,3-diaminobenzidine as chromogen (DAB; 0.5 mg/ml Sigma-Aldrich) [54].

### Image analysis

The total number of both Ki67 immunoreactive (IR) and BrdU-IR cells in the dentate gyrus was estimated using a modified version of the optical fractionator method on a systematic random sampling of every tenth section along the rostrocaudal axis of the hippocampal formation. Cell somata were identified and counted in both hemispheres, with a 40X objective using an Olympus BX51 light microscope linked to a computer. Cells appearing above and below the focal plane were omitted to prevent counting cell caps [54], [55].

The extent of the BLA lesions was measured through images from NeuN stained serial sections. The border of the amygdala delineating the BLA and the border of the lesion were manually traced and the area determined with Scion Image software (available on the web at http://www.scioncorp.com). Measurements were taken bilaterally along the entire rostrocaudal axis of the BLA (6–9 sections for each brain) and the final values represent the left–right average [56], [57].

### Statistical analyses

The SPSS 13.0 and Amos 17.0 (SPSS, Chicago, IL) statistical packages were used for the statistical analyses. Normality and homogeneity of variance was tested and adjusted statistics were used as required.

*Factorial analyses:* Factorial analyses were applied to characterize animals according to their anxiety-like behaviors from the EPM. A continuous, interval scale score was calculated through using principal components as extraction method and varimax rotation with Kaiser normalization rotation [58]. Individual factor scores were calculated for each subject through the relative weight and orientation (eigenvalues) of the parameters loading on the factor. Scores were generated using a Z distribution, where the value 0 corresponds to the mean, and values are expressed in terms of standard deviations. In addition, one of the SEM models performed was addressed to investigate differences due to extremes in anxiety values, which led us to classify animals in either low (LA) or highly (HA) anxious. Thus, a categorical dichotomous score was obtained by classifying subjects into groups of LA and HA, using the extreme lower and higher values of the continuous scores obtained from the factor analysis; subjects with scores within ±0.25 standard deviations from the mean were excluded from this classification.

*Parametric statistics:* The effect of BLA lesions on anxiety levels in the EPM were analyzed with a Student's *t*-test for independent samples. For the analyses of the impact of BLA lesions and anxiety on fluoxetine effects on indices of neurogenesis and depression-like behavior, there was the constraint that anxiety was affected by BLA lesions, and therefore it cannot be treated as an independent variable. Hence, instead of a three-way ANOVA, sequential two-way ANOVAs were used followed by simple effects analyses where appropriate. Analyses were performed according to a 2×2 design.

Since anxiety levels can be affected by BLA lesions [59], the relative contribution of lesion and anxiety on the behavioral and neurogenic effects of fluoxetine, cannot be addressed with ANOVAs. Therefore, we applied structural equation modeling (SEM) as a more flexible alternative statistical technique to achieve the stated aims of the current study.

*Structural equation modeling (SEM):* As a powerful extension of multiple regression analyses, SEM goes beyond bivariate correlations, to allow simultaneous tests of the contribution of multiple variables (numerical and categorical), both direct and indirect, and to establish their individual predictive ability over one or more dependent variables. Data were first examined to determine the suitability for multivariate analyses. The SEM analyses were based on the maximum likelihood estimation (MLE) and the following three indices of fit were used to test the ability of the proposed models to provide a plausible representation of the underlying variance structure of the data. In addition to a nonsignificant chi square (χ^2^), allowing a rejection of the null hypothesis (i.e. an unacceptable representation of the data), a good-fitting model was considered to have a comparative fit index (CFI) ≥0.95 and a root mean square approximation of variance (RMSEA) significantly smaller than 0.05 [60], [61].

In the figures for each of the proposed models, standardized beta weights along an arrow path indicate the ability of one variable to predict a dependent variable, ranging in absolute value from zero to one, and each with its statistical significance (p<0.05). The optimal model for each condition, including the path strengths between the tested variables were determined by first testing separately either level of each condition (lesion, drug treatment and anxiety level). Subsequently, a multi-group (“stacking”) approach was used to statistically compare model fit and path strengths between the groups. Here, an ‘inclusive’ model was tested, thus combining into one model effective paths from both levels of each condition. Next, each path was individually constrained to test for selective differences between the levels of each condition, e.g. [62]. Multi-group comparison statistics for stacked models specify the statistical significance of χ^2^ differences (Δχ^2^) when constrained and unconstrained models are compared. Using this approach, significant results indicate a meaningful difference in the strength of the path(s) tested between the groups (regardless of individual path statistical significance in either group). Furthermore, the squared multiple correlation (*R* ^2^ or coefficient of determination), representing a measure of the proportion of the variance of the dependent variable that is explained by the independent variables, is also presented.

## Results

### Verification of BLA lesions

The extent of the lesions for the rats included in the study is shown in Figure S1. Seventeen rats (out of the initial 43 lesioned rats) presenting an average lesion area smaller than 40% (criteria for exclusion) of the total BLA area were eliminated from the analyses of the study, which rendered an n = 26 in the BLA lesion group. As a consequence, the number of animals included in each experimental group was as follows: Sh/Vh (n = 17), Sh/Flx (n = 21), BLA/Vh (n = 12) and BLA/Flx (n = 14). As indicated in Figure S1, the minimum extent of the lesions always included at least 40% of the BLA while in a small percentage of cases the maximal lesion extension occupied adjacent medial and basal areas.

### Effects of BLA lesions on anxiety levels

A factor analysis applied to the behavioral parameters extracted from the EPM identified two main factors representing ‘anxiety’ and ‘locomotion’, with a value for each subject (Table S1). BLA lesions induced a significant reduction of anxiety levels as compared to sham treatment (*p*<0.01; Figure S2).

### Effects of BLA lesions and fluoxetine treatment on cell proliferation and survival and on depression-like behavior

**Figure 2:**
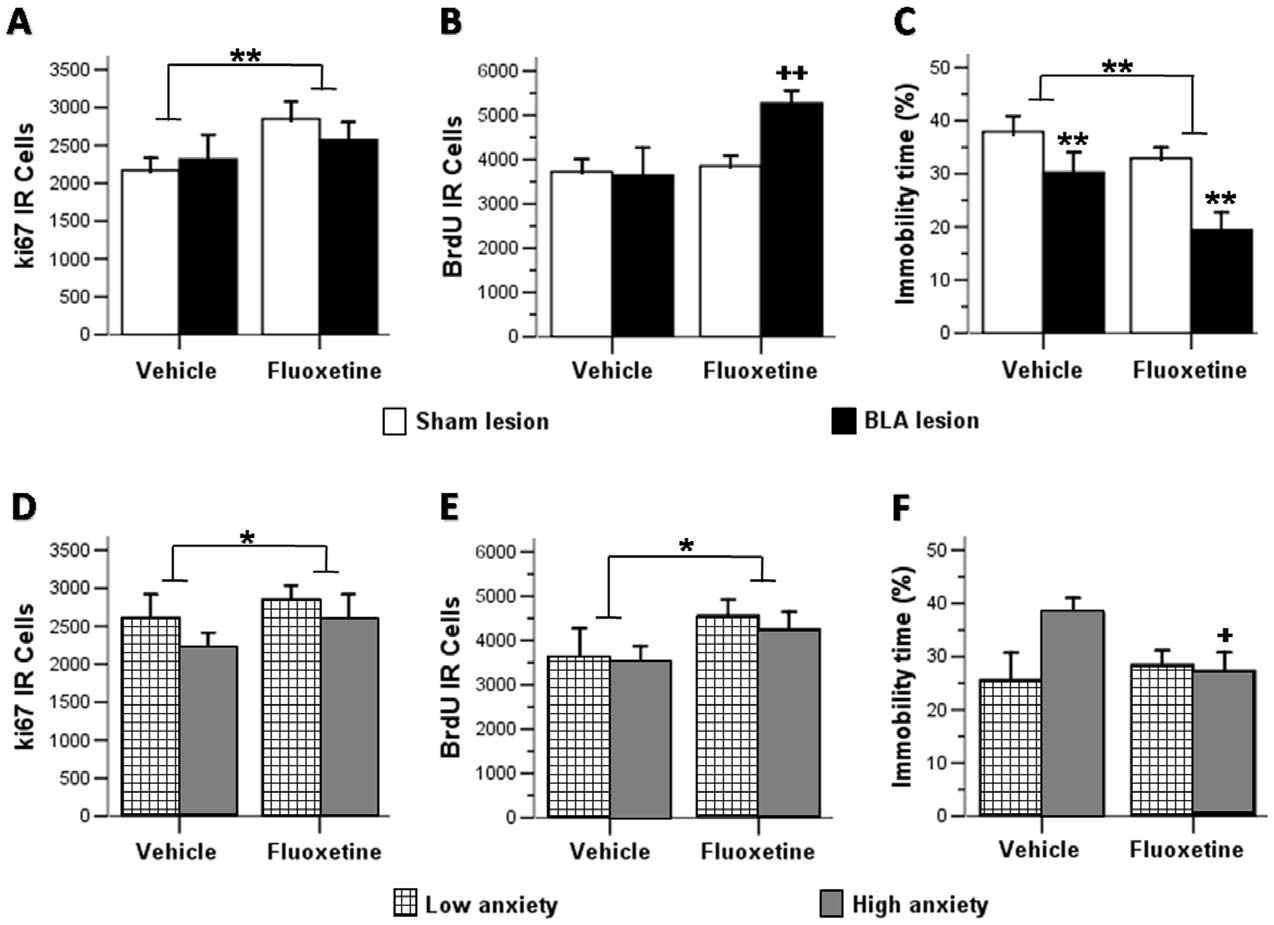
Effects of chronic treatment with fluoxetine on levels of cell proliferation and survival in the dentate gyrus of the hippocampus and depression-like behavior during the forced swimming test after neurochemical lesions of the basolateral complex of the amygdala (A,B,C) and according to anxiety (D,E,F). Error bars represent the standard error of the mean. [**p<0.01, *p<0.05 vs. corresponding vehicle group (ANOVA main effect); ++p<0.01, +p<0.05 vs. corresponding vehicle group (ANOVA simple main effect).

Independent two-way ANOVAs (BLA lesion and fluoxetine treatment as factors) were performed on indices of cell proliferation and survival and on the measure of depression-like behavior. A first ANOVA on cell proliferation data as indicated by the number of Ki67-IR cells in the DG, revealed a significant increase in Ki67-IR cells with fluoxetine (p<0.01), but no effect of BLA lesions (n.s.), and no significant interaction between the two factors (n.s.) (see section A in Figure 2). ANOVA on cell survival data as indicated by the number of BrdU-IR cells showed a significant interaction between lesion and drug treatment (p<0.05) (see section B in Figure 2), a simple effects analysis confirmed that fluoxetine treatment increased cell survival only in BLA lesioned rats (p<0.01).

ANOVA on the immobility time in the forced-swim test indicated a significant reduction with both lesion (p<0.01) and fluoxetine treatment (p<0.01), but no significant interaction (n.s.) (see section C in Figure 2).

### Effects of anxiety and fluoxetine treatment on cell proliferation and survival and on depression-like behavior

In order to study the impact of anxiety as a dichotomized variable, we classified subjects into groups of low (LA) and high (HA) anxiety by selecting those animals whose punctuation in the anxiety factor was 0.25 standard deviations either above (HA; n = 28; 19 sham y 9 lesion) or below (LA; n = 24; 11 sham and 13 lesion) the mean. Mean comparison confirmed that these two groups differed significantly on the anxiety factor (Student-t test; p<0.01; Figure S3). Then, independent two-way ANOVAs (anxiety levels and fluoxetine treatment as factors) were performed on indices of cell proliferation and survival and of depression-like behavior. Although ANOVAs performed respectively on Ki67- (see section D in Figure 2) and BrdU-IR (see section E in Figure 2) cell number indicated a lack of effect of anxiety on either of these indices of neurogenesis (all n.s.), a significant facilitating effect of fluoxetine treatment (all p<0.5) was observed. No significant interaction between these two factors was seen in either case.

Analysis of the behavioral outcome (see section F in Figure 2) indicated that high anxiety was significantly associated with prolonged immobility in the forced-swim test (p<0.05). While there was no effect of fluoxetine (n.s.), there was a significant interaction between the two factors (p<0.05), and a simple effects analysis confirmed that fluoxetine decreased immobility time only in the HA group (p<0.01).

### Correlational analyses between indices of anxiety, cell proliferation and survival and depression-like behavior

We then performed correlational analyses to address the following key questions: (i) whether differences in anxiety levels play a role in the studied effects, and (ii) whether there is a relationship between indices of neurogenesis and depression-like behavior. The analyses were performed on data from all animals in the study and then separately for each experimental group (see correlational matrix in the Table S2). Anxiety levels showed significant positive correlations with immobility in the forced-swim test both for the whole sample (r = 0.30, p<0.05) and for the BLA/Vh group (r = 0.79, p<0.01). Meanwhile negative correlations with Ki67-IR cell number were found in the same, BLA/Vh group (r = −0.77, p<0.01) and with BrdU-IR cells in the Sh/Vh group (r = −0.75, p<0.01). Exclusively for BLA/Vh animals was the immobility time in the forced-swim test negatively correlated with the levels of Ki67-IR cells (r = −0.67; p<0.01) and positively correlated with the levels of cell BrdU-IR cells (r = 0.75; p<0.05). For the other experimental groups, no significant correlations were observed.

### Structural equation modeling

Construction of the models was guided by the results from the ANOVAs and the correlational matrix (see above) on the relationships between the six studied variables (fluoxetine treatment, BLA lesion, anxiety levels, Ki67, BrdU, and FST immobility). Details for total and indirect effects and path strength differences for the presented models are shown in Tables S3 to S9. In the text, below, only significant relationships (and occasionally nearly significant trends) are indicated.

**Figure 3:**
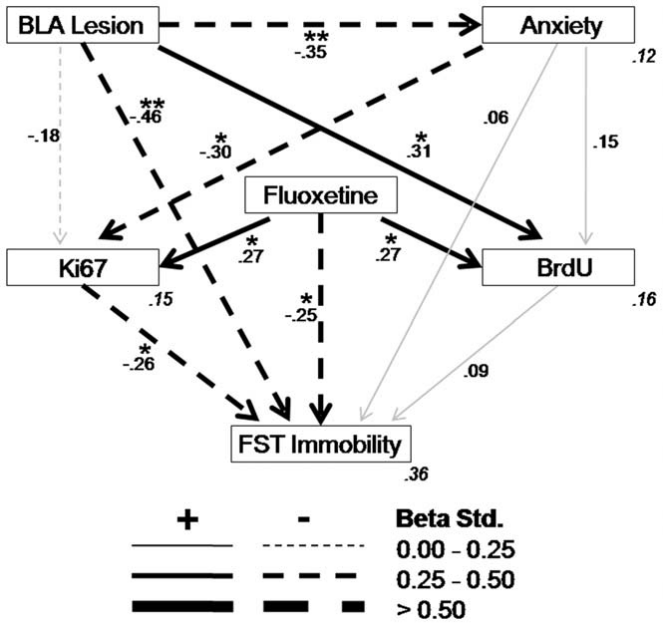
Path analysis and fit test for the general model (χ^2^ = 1.21, df = 2, p = 0.751; CFI = 1.00; RMSEA<0.01, p = 0.792). Significant paths (*p<0.05; **p<0.01) are in black and non significant in gray. The squared multiple correlations (*R* ^2^) of the endogenous variables are located in their right inferior corner in gray and italics.

A general model (Figure 3) was generated in which a relational structure between the variables was proposed to test the direct or indirect impact of each of the 3 predictor variables (fluoxetine treatment, BLA lesion, anxiety levels) on the 3 output measures (Ki67, BrdU, and FST immobility) and in turn of the neurogenic measures on the behavioral outcome. According to the criteria established, this is a good-fitting model (χ^2^ = 1.21, df = 2, p = 0.751; CFI = 1.00; RMSEA <0.01, p = 0.792, see Table S3 for total and indirect effects). As frequently reported in the literature, in this model including all animals and experimental conditions, we confirm similar positive effects of fluoxetine treatment on levels of both Ki67-IR cells and BrdU-IR cells, as well as a negative effect of fluoxetine on immobility time in the FST. We also observed that BLA lesions reduced anxiety levels and immobility time in the FST, while increasing the expression of BrdU-IR cells specifically. Anxiety levels were negatively related to levels of Ki67-IR cells, while the latter variable was in turn again negatively related to immobility time in the FST.

**Figure 4:**
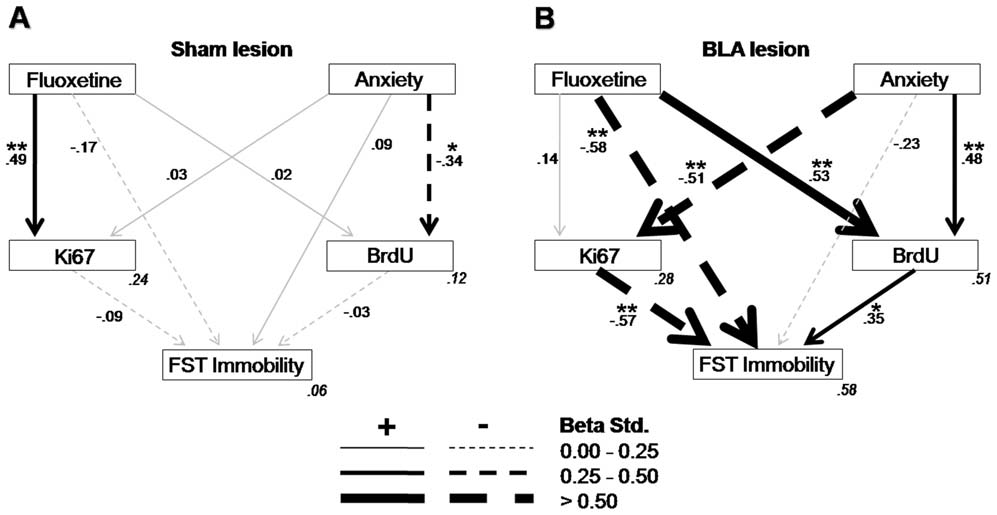
Path analysis for the models according to the lesion groups. **A.** Model fit was tested separately for the sham lesion group (χ^2^ = 5.42, df = 2, p = 0.066; CFI = 0.48; RMSEA = 0.21, p = 0.083) and **B.** for the BLA lesion group (χ^2^ = 0.52, df = 2, p = 0.771; CFI = 1.00; RMSEA<0.01, p = 0.783). Significant paths (p<0.05; **p<0.01) are in black and non significant in gray. The squared multiple correlations (*R* ^2^) of the endogenous variables are located in their right inferior corner in gray and italics.

Next, this general model was the basis to further assess whether the established relationships within the overall structure differed for each of the levels comprised in each of the three predicting variables (fluoxetine treatment, BLA lesion, anxiety levels). As a first step, three independent models were derived by excluding, in each case, one of the predicting variables. For each case, a good fitting model was obtained for at least one level (Figures 4–6), thus enabling multi-group stacked model analyses in order to directly compare both levels of the variable in question. Thus, specific path strength differences between the two levels of each variable could be statistically compared.

**Figure 5 pone-0008618-g005:**
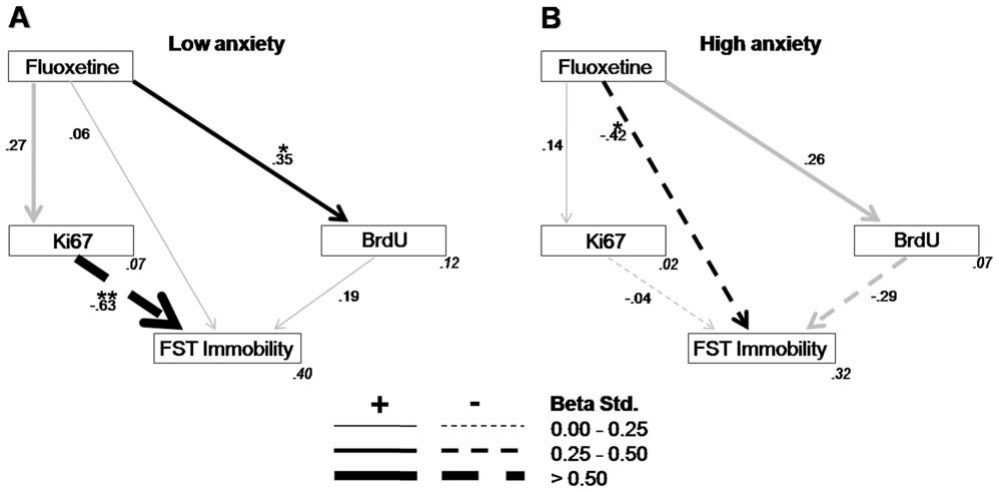
Path analysis for the models according to the anxiety groups. **A.** Model fit was tested separately for the low anxiety group (χ^2^ = 0.26, df = 1, p = 0.611; CFI = 1.00; RMSEA<0.01, p = 0.621) and **B.** for the high anxiety group (χ^2^ = 0.01, df = 1, p = 0.924; CFI = 1.00; RMSEA<0.01, p = 0.927). Significant paths (p<0.05; **p<0.01) are in black and non significant in gray. The squared multiple correlations (*R* ^2^) of the endogenous variables are located in their right inferior corner in gray and italics.

**Figure 6:**
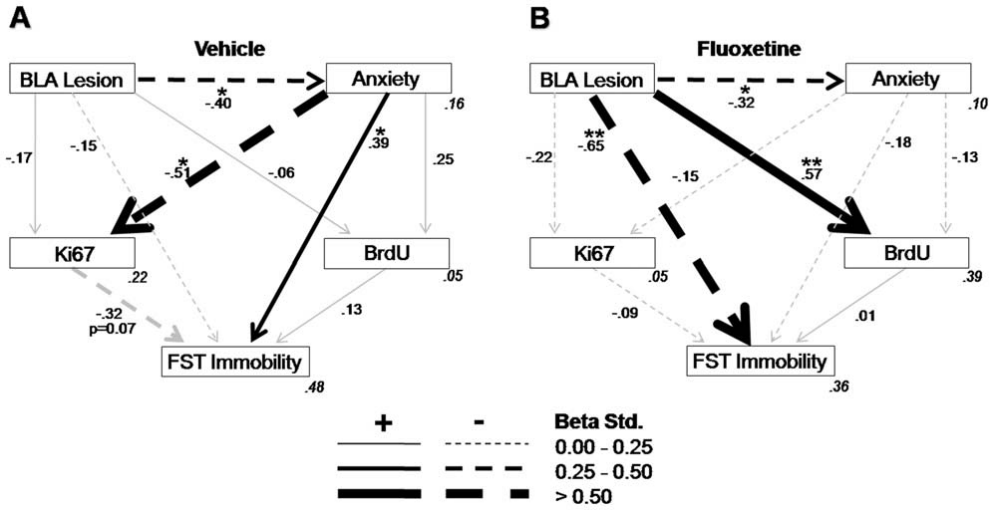
Path analysis for the models according to the drug treatment groups. **A.** Model fit was tested separately for the vehicle group (χ^2^ = 0.07, df = 1, p = 0.784; CFI = 1.00; RMSEA<0.01, p = 0.791) and **B.** for the fluoxetine group (χ^2^ = 2.00, df = 1, p = 0.157; CFI = 0.94; RMSEA = 0.17, p = 0.175). Significant paths (p<0.05; **p<0.01) are in black and non significant in gray. The squared multiple correlations (*R* ^2^) of the endogenous variables are located in their right inferior corner in gray and italics.

*Path analyses for the modulatory role of BLA lesions:* To assess the potential modulation of BLA lesions over the neurogenic and behavioral actions of fluoxetine, a stacked, multi-sample analysis was used to compare Sham and BLA lesion groups (Figure 4). Importantly, stacked group analyses revealed an overall difference in the model fit between the sham and the BLA lesion groups (p<0.002, Table S4). This analysis also confirmed the existence of significant differences between Sham and BLA lesioned groups, as exhibited by the beta weights shown in Figure 4, specifically in three pathways: (i) the effect of fluoxetine treatment on BrdU-IR cells, reflecting the fact that in the BLA lesion condition, fluoxetine treatment was related to higher levels of BrdU-IR cells, a relationship that was absent in the sham lesion condition; (ii) the relation between anxiety and BrdU-IR cells: whereas anxiety predicted lower levels of BrdU-IR cells in the sham lesion condition, an opposite pattern was observed for BLA lesioned animals; (iii) the relationship between Ki67 levels and immobility in the FST, with this relationship been negative for BLA lesioned animals but inexistent in the sham group.

In addition, it may be worth noting other pathways in which differences among the two groups approached significance. In all the following cases a relationship present in the BLA lesion group was notably weaker, if not absent, than in the intact BLA group: (i) a negative influence of fluoxetine in FST immobility; (ii) a positive relationship of BrdU on FST immobility; and (iii) a negative influence of anxiety in Ki67. Furthermore, as the beta weights show in Figure 4, specifically in the BLA lesion group, anxiety *indirectly* related to increased FST immobility via opposite regulation of Ki67 and BrdU (total and indirect effect breakdown, see Table S5). This BLA lesion model exhibited particularly high predictive power (*R* ^2^ values in Fig. 4B). The joint contribution of anxiety and fluoxetine accounted for 51% of the variance of BrdU levels; and fluoxetine with anxiety, the latter indirectly through both measures of neurogenic activity (Ki67 and BrdU), also in turn successfully accounted for an important proportion of the variance of the immobility measure (58%).

*Path analyses for the modulatory role of anxiety:* In order to test the potential modulatory role of anxiety levels over the neurogenic and behavioral actions of fluoxetine, a multiple-sample analysis was generated for the low and high anxiety groups. In this case, because most BLA lesioned subjects also exhibited low anxiety, thus yielding uneven groups precluding formal statistical comparisons, the BLA factor was excluded. The resulting model revealed a significant overall difference in model fit between low and high anxiety groups (Figure 5; p<0.01, Table S6). A main significant difference between the anxiety groups was the influence of Ki67 on FST immobility, which exhibited a strong negative relationship in the LA group, while being absent in the HA group. Additionally, in spite of their weak strength, the opposite relationships between BrdU and FST immobility was also significant. It is finally worth noting the path from fluoxetine to FST immobility was negative in HA animals but absent in the LA group, a difference that approached significance (Table S6). For total and indirect effects see Table S7.

A final stacked multi-sample model (Figure 6; Table S8) was considered next to complement the above findings, specifically addressing differences due to the drug treatment. There was an overall difference in the model fit between vehicle and fluoxetine treated groups (p<0.05). A main difference between the drug treatments was the way that, compared to vehicle, treatment with fluoxetine reduced the relationship from anxiety to FST immobility (p<0.05). Additionally, an increase in the negative relationship of BLA lesion to FST immobility approached significance (p = 0.066). Finally, as the beta weights show in Figure 6, specifically in the Vehicle group, the BLA lesion indirectly increased Ki67 levels by down-regulating anxiety (Table S9).

Overall, in addition to the confirmation of the result obtained with the univariate analyses that fluoxetine treatment had a positive effect on BrdU-IR only when the BLA was lesioned, the main findings of the path analyses, that could not have been predicted from the univariate analyses, are the revelation that i) anxiety was critically related to BrdU levels, and this relationship varied with the lesion condition, being negative in sham animals and positive in BLA lesioned ones; and (ii) BLA lesions and low anxiety were critical factors to facilitate a negative relationship between cell proliferation and depression-like behavior.

## Discussion

Our goal here was to investigate whether the amygdala plays a key role on fluoxetine modulation of the proliferation and survival of progenitor cells in the dentate gyrus of the adult hippocampus, and on its impact on an index of depression-like behavior. A lesion approach was applied to specifically study the contribution of the BLA. Since BLA lesions affected animals' anxiety, and emerging evidence indicates that anxiety trait is linked to hippocampal neurogenesis [43], [51], [63], structural equation modeling (SEM) was applied to assess the relative contribution of both BLA lesions and anxiety on fluoxetine effects. In addition, we explored the relationship of indices of cell proliferation and survival to the selected index of depression-like behavior (i.e., immobility in the forced swim test).

We found evidence in sham-lesioned rats of a facilitating effect of chronic fluoxetine on cell proliferation, but not on cell survival, as recently reported in adult male rats [64]. The increase in cell proliferation [4], [65], [66] but see and lack of effect on cell survival [67], [68] are in agreement with previous findings in the literature. See [69] for negative effects on cell proliferation and [4] for positive effects on cell survival.

Both parametric statistics and SEM confirmed a key role for BLA lesions in enabling a facilitating effect of fluoxetine on the survival of recently generated cells, suggesting that under non lesion conditions, the BLA restrains the positive effects of the antidepressant on cell survival (Fig. 4). As a note of caution, it is important to notice that although in all cases amygdala lesions included at least 40% of the BLA, in some cases the maximum extension included adjacent basal and/or medial areas whose potential role in the present observations cannot be excluded. Interestingly, these results implicating the involvement of the BLA on hippocampal cell survival stimulated by fluoxetine, but not under basal conditions, are reminiscent of the BLA involvement in the modulation of memory consolidation in other brain regions (notably the hippocampus) induced by stress and emotions without affecting memory formation in itself [32], [36], [70]. A potential role for the amygdala in these results is supported by several examples of inhibitory effects of SSRIs on amygdala activity in animals [19], [22] and humans as well [23], although activating effects were also reported [71], as evidence indicating that enhanced amygdala activity as induced by seizures associated with rapid electrical amygdala kindling increases the addition of newly generated granule cell neurons to the granular cell layer of the dentate gyrus [72]. Alternatively, the BLA might be exerting a modulatory influence on the hippocampal *milieu* that is relevant for fluoxetine-stimulated cell survival. This concept is linked to the view that the amygdala is able to affect both the induction of plasticity [73], [74] and meta-plasticity [35] in the dentate gyrus. Translated into the current framework, amygdala influences on cell survival might be achieved by BLA modulation of fluoxetine's molecular and network targets in the hippocampus.

The stacked model (Fig. 4) revealed a surprising relationship between anxiety and cell survival that differed upon the lesion condition: while in Sham animals anxiety levels negatively predicted cell survival, this relationship was positive in BLA lesioned rats. Given the strong anxiolytic effects induced by BLA lesions, these results reveal the existence of an inverted-U shape relationship between anxiety and cell survival, with both low and high extremes in anxiety being related to lower levels of cell survival than intermediate levels of anxiety. Although intriguing given its novelty in the field of neurogenesis, the existence of an inverted-U shape between emotionality and hippocampal function is not surprising. There are many examples in the literature for complex effects of stress on cognition [75], [76], [77], [78], [79] and hippocampal plasticity [80], [81] showing similar biphasic relationships. Further studies should explore to what extent this observation for an inverted-U shape between anxiety and neurogenesis is applicable to the range of normal variation in anxiety trait or related, for the lower extreme of the anxiety spectrum to pathological conditions linked to amygdala damage.

As for cell proliferation, our analyses in contrast revealed that BLA lesions seem to indirectly influence its rate through their effects in anxiety: by reducing anxiety levels, which are negatively related to cell proliferation, BLA lesions seem to have a positive impact in cell proliferation. In fact, anxiety on its own was a highly predictive factor of cell proliferation. This finding is in agreement with emerging evidence supporting a link between anxiety and hippocampal neurogenesis. Individual differences in rats' trait anxiety were related to the negative and positive impact, respectively, of chronic stress and chronic treatment with a corticotrophin releasing factor receptor 1 (CRFR1) antagonist on hippocampal cell proliferation [51]. Transgenic mice, in which hippocampal neurogenesis was specifically impaired, exhibited a striking increase in anxiety-related behaviors [43]. It should also be noted that several studies did not find any impact of inhibiting neurogenesis *on* anxiety- or depression-related behaviors [9], [82], [83], [84].

We also found that BLA lesions exerted antidepressive behavioral effects irrespective of fluoxetine treatment. These results are in agreement with previous observations showing attenuated anxiety- and depression-related behaviors following bilateral BLA lesions in animals [85] and humans [86], while anxiogenic-like effects after amygdala stimulation through kindling protocols [87]. Viral-induced increase in the molecular expression of the transcription factor CREB in the BLA in rats was also shown to result in changes in anxiety- and depression-like behaviors [88]. Moreover, anxiety here largely explained (partly via Ki67, and also via other unspecified factors) differences in depression-like behavior in vehicle treated animals. Note that anxiety was evaluated before drug treatment and that anxiety levels have been reported to be changed by fluoxetine [18], [89]. Interestingly, these results are also in line with a large body of literature in humans indicating close links between high anxiety trait, depression and the propensity of the amygdala to show hyperactivation [42].

A specific goal of this study was to explore whether BLA lesions and anxiety might influence the link from hippocampal cell proliferation and survival to depression-like behavior, a topic that has received a great deal of attention in recent years. A view is emerging that pre-existing anxiety might determine the range of behavioral effects displayed by chronic antidepressant treatment [9], [69], [90]. Our SEM analyses suggest that, in relation to the attenuation of depression-like behavior, reduced BLA output and low anxiety are critical factors determining the engagement of newborn cells—differentially affecting proliferative potential and survival. These findings might help explain existing discrepancies in the literature with regards to the dependence of behavioral effects of antidepressants on hippocampal neurogenesis [3]. In fact, antidepressant-like behavioral effects of chronic fluoxetine treatment were blocked by ablation of neurogenesis in the low anxious strain of mice, C57BL/6 [9], but not in the highly anxious strain BABL/c [91]. Although neurogenesis is frequently found to be unnecessary for fluoxetine effects in the forced-swim test in mice [8], [91], it was shown to be critical in rats [92], supporting the specific link observed in our study. However, it is important to note that a recent study showed that antidepressants (including fluoxetine) retain their therapeutic efficacy in reducing both several indices of depression-like behavior (including the forced swim test) even when neurogenesis is pharmacologically blocked implicating, instead neuronal remodeling as the mechanism associated with the antidepressant activity [93].

Anxiety seems to be central to the converging effects of antidepressant treatment and BLA lesions. It emerged that modulation of cell proliferation by fluoxetine was particularly strong with a normally functioning BLA. Critically, the antidepressant treatment was found to suppress the inhibitory indirect effect of the BLA by reducing the impact of anxiety on cell proliferation (Fig. 6). However, the relationship between anxiety and neurogenesis is still far from being clearly understood. A recent report showed that administration of the anxiolytic diazepam simultaneously to fluoxetine treatment completely blocked the SSRI-induced increase in both neurogenesis and cell survival [94].

Taken together, our observations would suggest that the behavioral effects of antidepressants seem to be antagonized by a normally functioning BLA. Furthermore, antidepressants and normal BLA function exert opposite, interacting influences over cell proliferation and survival that is largely associated with the resulting impact of anxiety (Fig. 3).

In summary, our study highlights an important role for the amygdala on fluoxetine-stimulated cell proliferation and survival, as well as on the establishment of a link between cell proliferation and depression-like behavior. It also highlights an important role for anxiety in mediating the effects of basolateral amygdala and antidepressant activity on cell proliferation and survival. The application of structural equation modeling in the investigation of a complex, multidimensional phenomenon allowed us to capture important contributing factors operating together to determine the behavioral and neurobiological outcomes of fluoxetine treatment. Given the multiple targets of antidepressants effects in the brain [95], [96], our study emphasizes the importance of considering indirect actions involved in their modulatory effects on adult hippocampal neurogenesis.

## Supporting Information

Figure S1 BLA histochemical lesions with NMDA. A. Reconstruction of the minimal (black) and maximal (gray) extents of lesion. B. NeuN immunohistochemistry for representative BLA lesions. C. NeuN immunohistochemistry for representative sham lesion. Coordinates of the coronal sections are indicated with reference to Bregma. Plates are adapted from the atlas of Paxinos and Watson (Paxinos and Watson, 1997).
(1.80 MB TIF)

Figure S2 Effect of BLA neurochemical lesion on anxiety levels. Values show the mean + SEM. **p<0.01.
(0.37 MB TIF)

Figure S3 Levels of anxiety after classification of animals into the dichotomized categories of low and high anxiety. Values show the mean + SEM. **p<0.01.
(0.43 MB TIF)

Table S1 Factor analysis for the Elevated Plus Maze.
(0.03 MB DOC)

Table S2 Correlation matrix for anxiety levels, cell proliferation, cell survival, and depression-like behavior in the total sample and by groups of lesion and drug treatment.
(0.05 MB DOC)

Table S3 Total and indirect effects for the general model in Figure 3.
(0.03 MB DOC)

Table S4 Multiple-sample structural equation model analyses as shown in Figure 4.
(0.04 MB DOC)

Table S5 Total and indirect effects for models in Figure 4.
(0.04 MB DOC)

Table S6 Multiple-sample structural equation model analyses as shown in Figure 5.
(0.03 MB DOC)

Table S7 Total and indirect effects for models in Figure 5.
(0.03 MB DOC)

Table S8 Multiple-sample structural equation model analyses as shown in Figure 6.
(0.04 MB DOC)

Table S9 Total and indirect effects for models in Figure 6.
(0.04 MB DOC)
