# Supplementary figures and images for: Role of the Amygdala in Antidepressant Effects on Hippocampal Cell Proliferation and Survival and on Depression-like Behavior in the Rat
Source: PLoS One. 2010 Jan 8;5(1):e8618. doi: 10.1371/journal.pone.0008618 (PMC2799663; doi:10.1371/journal.pone.0008618)

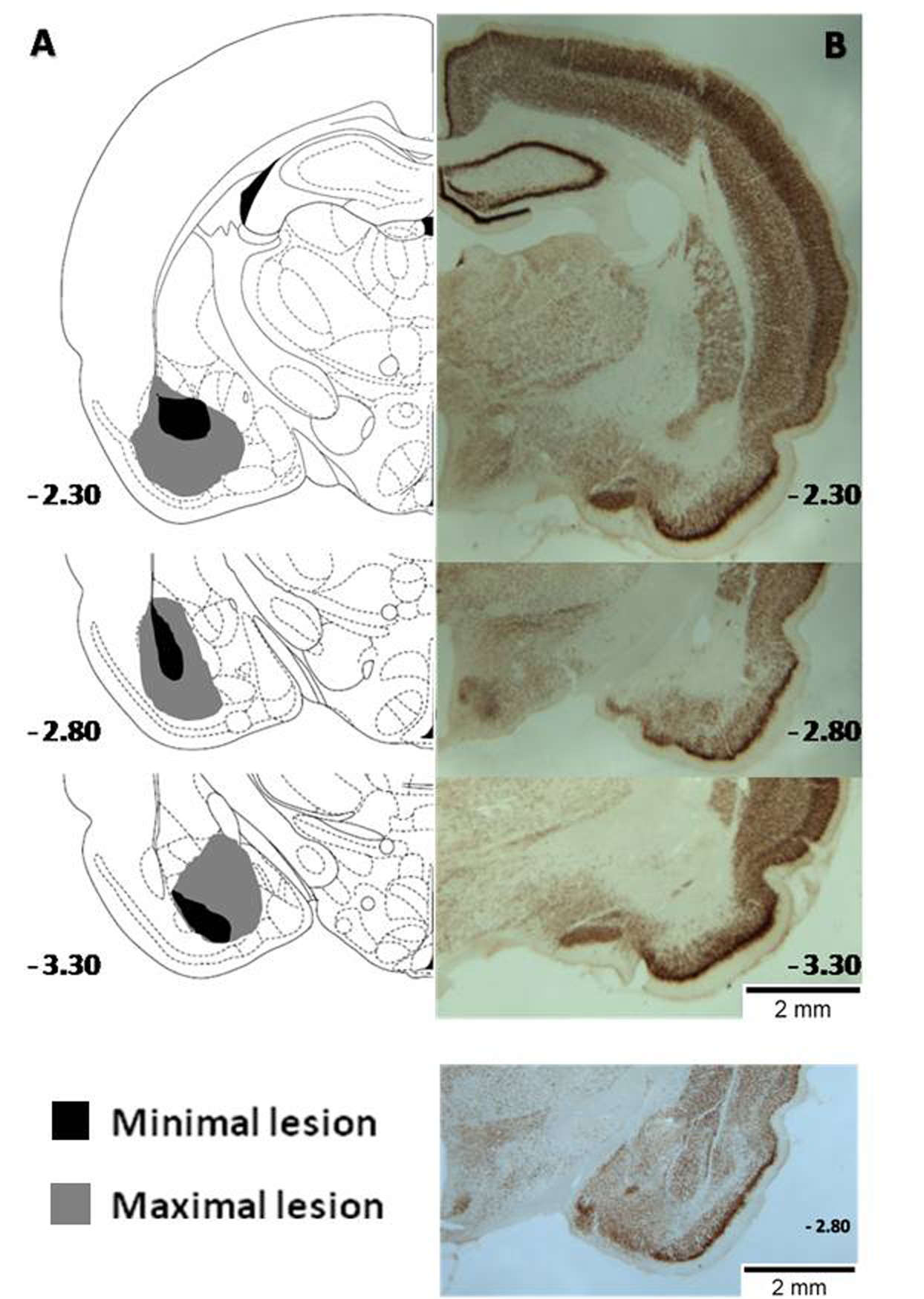

Supplement: Figure S1 — BLA histochemical lesions with NMDA. A. Reconstruction of the minimal (black) and maximal (gray) extents of lesion. B. NeuN immunohistochemistry for representative BLA lesions. C. NeuN immunohistochemistry for representative sham lesion. Coordinates of the coronal sections are indicated with reference to Bregma. Plates are adapted from the atlas of Paxinos and Watson (Paxinos and Watson, 1997). (1.80 MB TIF) [file pone.0008618.s001.tif]

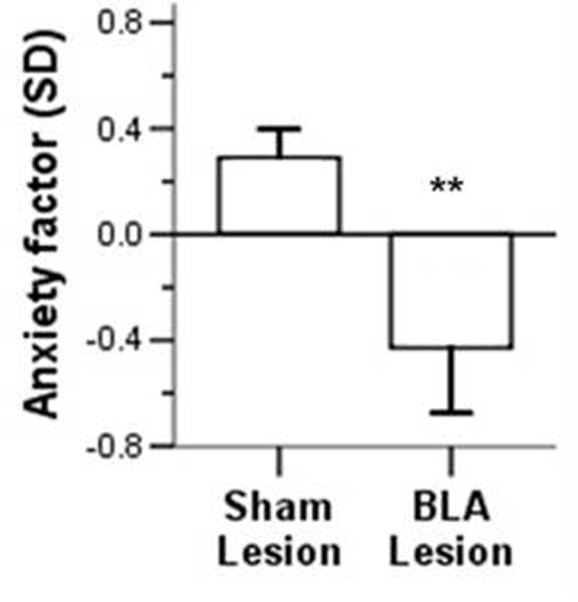

Supplement: Figure S2 — Effect of BLA neurochemical lesion on anxiety levels. Values show the mean + SEM. **p<0.01. (0.37 MB TIF) [file pone.0008618.s002.tif]

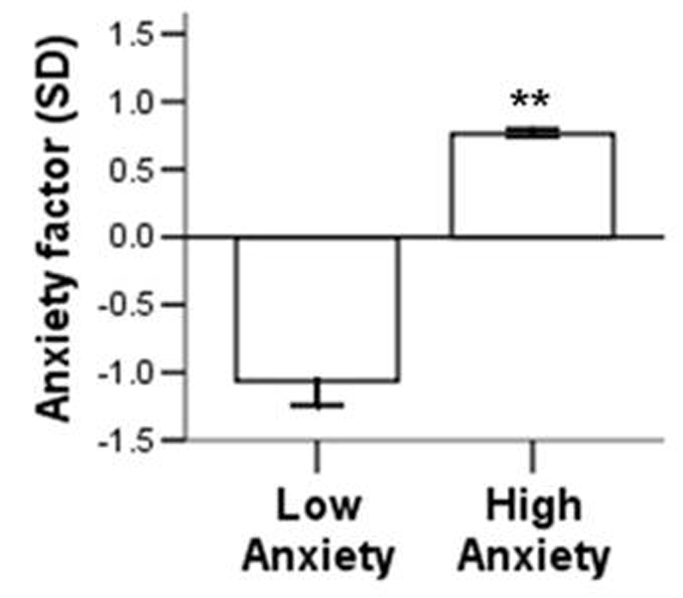

Supplement: Figure S3 — Levels of anxiety after classification of animals into the dichotomized categories of low and high anxiety. Values show the mean + SEM. **p<0.01. (0.43 MB TIF) [file pone.0008618.s003.tif]
