# Supplementary material for: Role of the Amygdala in Antidepressant Effects on Hippocampal Cell Proliferation and Survival and on Depression-like Behavior in the Rat
Source: PLoS One. 2010 Jan 8;5(1):e8618. doi: 10.1371/journal.pone.0008618 (PMC2799663; doi:10.1371/journal.pone.0008618)
Supplement: Table S1 — Factor analysis for the Elevated Plus Maze. (0.03 MB DOC) [file pone.0008618.s004.doc]

| Behavioral parameters | Anxiety  trait | Locomotion  trait | % of Variance | KMO | Bartlett |
| --- | --- | --- | --- | --- | --- |
| Distance open arms | **.952** | -.012 |  |  |  |
| Time in open arms | **.959** | -.037 |  |  |  |
| Time in closed arms | **-.758** | -.407 | 83.84 | 0.57 | p < 0.01 |
| Distance close arms | -.097 | **.876** |  |  |  |
| Transitions close arms | .236 | **.899** |  |  |  |

**Table S1.** Factor analysis for the Elevated Plus Maze
