# Supplementary material for: Role of the Amygdala in Antidepressant Effects on Hippocampal Cell Proliferation and Survival and on Depression-like Behavior in the Rat
Source: PLoS One. 2010 Jan 8;5(1):e8618. doi: 10.1371/journal.pone.0008618 (PMC2799663; doi:10.1371/journal.pone.0008618)
Supplement: Table S2 — Correlation matrix for anxiety levels, cell proliferation, cell survival, and depression-like behavior in the total sample and by groups of lesion and drug treatment. (0.05 MB DOC) [file pone.0008618.s005.doc]

**Table S2.** Correlation matrix for anxiety levels, cell proliferation, cell survival and depression-like behavior in the total sample and by groups of lesion and drug treatment

|  | Group | Anxiety | Ki67 | BrdU |
| --- | --- | --- | --- | --- |
| Ki67 | All | - 0.27 |  |  |
|  | Sh/Vh | 0.37 |  |  |
|  | Sh/Flx | - 0.08 |  |  |
|  | BLA/Vh | - 0.77** |  |  |
|  | BLA/Flx | - 0.26 |  |  |
| BrdU | All | 0.07 | 0.09 |  |
|  | Sh/Vh | - 0.75** | - 0.05 |  |
|  | Sh/Flx | - 0.26 | 0.57* |  |
|  | BLA/Vh | 0.59 | - 0.32 |  |
|  | BLA/Flx | 0.14 | - 0.20 |  |
| FST Immobility | All | 0.30* | - 0.28 | - 0.08 |
|  | Sh/Vh |  | - 0.41 | - 0.32 |
|  | Sh/Flx | 0.10 | - 0.12 | - 0.13 |
|  | BLA/Vh | 0.79** | - 0.67** | 0.75* |
|  | BLA/Flx | - 0.45 | - 0.11 | 0.23 |
| * p < 0.05 | ** p < 0.01 |  |  |  |
