# Supplementary material for: Role of the Amygdala in Antidepressant Effects on Hippocampal Cell Proliferation and Survival and on Depression-like Behavior in the Rat
Source: PLoS One. 2010 Jan 8;5(1):e8618. doi: 10.1371/journal.pone.0008618 (PMC2799663; doi:10.1371/journal.pone.0008618)
Supplement: Table S3 — Total and indirect effects for the general model in Figure 3. (0.03 MB DOC) [file pone.0008618.s006.doc]

|  | BLA Lesion |  | Fluoxetine |  | Anxiety |  | Ki67 |  | BrdU |  |
| --- | --- | --- | --- | --- | --- | --- | --- | --- | --- | --- |
|  | Total | Indirect | Total | Indirect | Total | Indirect | Total | Indirect | Total | Indirect |
| Anxiety | - 0.35 | 0.00 |  |  |  |  |  |  |  |  |
| Ki67 | - 0.07 | 0.10 | 0.27 | 0.00 | - 0.30 | 0.00 |  |  |  |  |
| BrdU | 0.26 | - 0.05 | 0.27 | 0.00 | 0.15 | 0.00 |  |  |  |  |
| FST Immobility | - 0.44 | - 0.02 | - 0.29 | - 0.04 | 0.15 | 0.09 | - 0.26 | 0.00 | - 0.09 | 0.00 |

**Table S3.** Total and indirect effects for the general model in Figure 3
