# Supplementary material for: Role of the Amygdala in Antidepressant Effects on Hippocampal Cell Proliferation and Survival and on Depression-like Behavior in the Rat
Source: PLoS One. 2010 Jan 8;5(1):e8618. doi: 10.1371/journal.pone.0008618 (PMC2799663; doi:10.1371/journal.pone.0008618)
Supplement: Table S4 — Multiple-sample structural equation model analyses as shown in Figure 4. (0.04 MB DOC) [file pone.0008618.s007.doc]

**Table S4.** Multiple-sample structural equation model analyses as shown in Figure 4

| Sham lesion VS BLA lesion | df | ∆x² | p |
| --- | --- | --- | --- |
| All paths | 3 | 14.55 | 0.002 |
| Fluoxetine to BrdU | 1 | 9.78 | 0.002 |
| Fluoxetine to Ki67 | 1 | 2.59 | 0.108 |
| Fluoxetine to FST Immobility | 1 | 3.49 | 0.062 |
| Anxiety to BrdU | 1 | 10.78 | 0.001 |
| Anxiety to Ki67 | 1 | 2.79 | 0.095 |
| Anxiety to FST Immobility | 1 | 1.43 | 0.232 |
| BrdU to FST Immobility | 1 | 3.24 | 0.063 |
| Ki67 to FST Immobility | 1 | 6.07 | 0.014 |
