# Supplementary material for: Role of the Amygdala in Antidepressant Effects on Hippocampal Cell Proliferation and Survival and on Depression-like Behavior in the Rat
Source: PLoS One. 2010 Jan 8;5(1):e8618. doi: 10.1371/journal.pone.0008618 (PMC2799663; doi:10.1371/journal.pone.0008618)
Supplement: Table S5 — Total and indirect effects for models in Figure 4. (0.04 MB DOC) [file pone.0008618.s008.doc]

**Table S5.** Total and indirect effects for models in Figure 4

|  | Anxiety |  | Fluoxetine |  | FST Immobility |  |
| --- | --- | --- | --- | --- | --- | --- |
|  | Total | Indirect | Total | Indirect | Total | Indirect |
| Sham lesion group |  |  |  |  |  |  |
| FST Immobility | 0.10 | 0.01 | - 0.22 | - 0.04 |  |  |
| Ki67 | 0.03 | 0.00 | 0.49 | 0.00 | - 0.09 | 0.00 |
| BrdU | - 0.34 | 0.00 | 0.02 | 0.00 | - 0.03 | 0.00 |
| BLA lesion group |  |  |  |  |  |  |
| FST Immobility | **0.23** | **0.46** | - 0.48 | 0.10 |  |  |
| Ki67 | - 0.51 | 0.00 | 0.14 | 0.00 | - 0.57 | 0.00 |
| BrdU | 0.48 | 0.00 | 0.53 | 0.00 | 0.35 | 0.00 |
