# Supplementary material for: Role of the Amygdala in Antidepressant Effects on Hippocampal Cell Proliferation and Survival and on Depression-like Behavior in the Rat
Source: PLoS One. 2010 Jan 8;5(1):e8618. doi: 10.1371/journal.pone.0008618 (PMC2799663; doi:10.1371/journal.pone.0008618)
Supplement: Table S6 — Multiple-sample structural equation model analyses as shown in Figure 5. (0.03 MB DOC) [file pone.0008618.s009.doc]

**Table S6.** Multiple-sample structural equation model analyses as shown in Figure 5

| Low Anxiety VS High Anxiety | df | ∆x² | p |
| --- | --- | --- | --- |
| All paths | 5 | 13.77 | 0.017 |
| Fluoxetine to BrdU | 1 | 0.29 | 0.589 |
| Fluoxetine to Ki67 | 1 | 0.50 | 0.481 |
| Fluoxetine to FST Immobility | 1 | 2.88 | 0.090 |
| BrdU to FST Immobility | 1 | 4.31 | 0.038 |
| Ki67 to FST Immobility | 1 | 11.32 | 0.001 |
