# Supplementary material for: Role of the Amygdala in Antidepressant Effects on Hippocampal Cell Proliferation and Survival and on Depression-like Behavior in the Rat
Source: PLoS One. 2010 Jan 8;5(1):e8618. doi: 10.1371/journal.pone.0008618 (PMC2799663; doi:10.1371/journal.pone.0008618)
Supplement: Table S7 — Total and indirect effects for models in Figure 5. (0.03 MB DOC) [file pone.0008618.s010.doc]

**Table S7.** Total and indirect effects for models in Figure 5

|  | Fluoxetine |  | Ki67 |  | BrdU |  |
| --- | --- | --- | --- | --- | --- | --- |
|  | Total | Indirect | Total | Indirect | Total | Indirect |
| Low Anxiety group |  |  |  |  |  |  |
| Ki67 | 0.27 | 0.00 |  |  |  |  |
| BrdU | 0.34 | 0.00 |  |  |  |  |
| FST Immobility | - 0.05 | - 0.11 | - 0.63 | 0.00 | 0.19 | 0.00 |
| High Anxiety group |  |  |  |  |  |  |
| Ki67 | 0.14 | 0.00 |  |  |  |  |
| BrdU | 0.26 | 0.00 |  |  |  |  |
| FST Immobility | - 0.49 | - 0.07 | 0.04 | 0.00 | - 0.29 | 0.00 |
