# Supplementary material for: Role of the Amygdala in Antidepressant Effects on Hippocampal Cell Proliferation and Survival and on Depression-like Behavior in the Rat
Source: PLoS One. 2010 Jan 8;5(1):e8618. doi: 10.1371/journal.pone.0008618 (PMC2799663; doi:10.1371/journal.pone.0008618)
Supplement: Table S8 — Multiple-sample structural equation model analyses as shown in Figure 6. (0.04 MB DOC) [file pone.0008618.s011.doc]

**Table S8.** Multiple-sample structural equation model analyses as shown in Figure 6

| Vehicle VS Fluoxetine | df | ∆x² | p |
| --- | --- | --- | --- |
| All paths | 9 | 20.11 | 0.017 |
| Lesion to Anxiety | 1 | 0.04 | 0.836 |
| Lesion to Ki67 | 1 | 0.05 | 0.819 |
| Lesion to BrdU | 1 | 2.89 | 0.089 |
| Lesion to FST Immobility | 1 | 3.38 | 0.066 |
| Anxiety to Ki67 | 1 | 1.55 | 0.213 |
| Anxiety to BrdU | 1 | 1.74 | 0.186 |
| Anxiety to FST Immobility | 1 | 5.28 | 0.022 |
| Ki67 to FST Immobility | 1 | 1.01 | 0.315 |
| BrdU to FST Immobility | 1 | 0.18 | 0.668 |
