# Supplementary material for: Role of the Amygdala in Antidepressant Effects on Hippocampal Cell Proliferation and Survival and on Depression-like Behavior in the Rat
Source: PLoS One. 2010 Jan 8;5(1):e8618. doi: 10.1371/journal.pone.0008618 (PMC2799663; doi:10.1371/journal.pone.0008618)
Supplement: Table S9 — Total and indirect effects for models in Figure 6. (0.04 MB DOC) [file pone.0008618.s012.doc]

|  | BLA Lesion |  | Anxiety |  | Ki67 |  | BrdU |  |
| --- | --- | --- | --- | --- | --- | --- | --- | --- |
|  | Total | Indirect | Total | Indirect | Total | Indirect | Total | Indirect |
| Vehicle group |  |  |  |  |  |  |  |  |
| Anxiety | - 0.40 | 0.00 |  |  |  |  |  |  |
| Ki67 | **0.03** | **0.20** | - 0.51 | 0.00 |  |  |  |  |
| BrdU | - 0.04 | - 0.10 | 0.25 | 0.00 |  |  |  |  |
| FST Immobility | - 0.32 | - 0.17 | 0.58 | 0.19 | - 0.32 | 0.00 | 0.12 | 0.00 |
| Fluoxetine group |  |  |  |  |  |  |  |  |
| Anxiety | - 0.32 | 0.00 |  |  |  |  |  |  |
| Ki67 | - 0.17 | 0.05 | - 0.15 | 0.00 |  |  |  |  |
| BrdU | 0.61 | 0.04 | - 0.12 | 0.00 |  |  |  |  |
| FST Immobility | - 0.57 | 0.08 | - 0.16 | 0.01 | - 0.09 | 0.00 | 0.01 | 0.00 |

**Table S9.** Total and indirect effects for models in figure 6
